# Tracing the genetic history of the ‘Cañaris’ from Ecuador and Peru using uniparental DNA markers

**DOI:** 10.1186/s12864-020-06834-1

**Published:** 2020-09-10

**Authors:** José R. Sandoval, Daniela R. Lacerda, Marilza M. S. Jota, Paulo Robles-Ruiz, Pierina Danos, César Paz-y-Miño, Spencer Wells, Fabrício R. Santos, Ricardo Fujita

**Affiliations:** 1 Centro de Investigación de Genética y Biología Molecular (CIGBM), Instituto de Investigación, Facultad de Medicina, Universidad de San Martín de Porres, Lima, Peru; 2 Laboratório de Biodiversidade e Evolução Molecular (LBEM), Instituto de Ciências Biológicas, Universidade Federal de Minas Gerais, Belo Horizonte, Brazil; 3 Instituto de Investigaciones Biomédicas, Universidad de las Américas, Quito, Ecuador; 4 Centro de Investigación Genética y Genómica, Universidad Tecnológica Equinoccial, Quito, Ecuador; 5 Department of Integrative Biology, University of Texas at Austin, Austin, TX USA

**Keywords:** Population genetics, Cañaris, SNP, Short tandem repeats, Y chromosome, Mitochondrial DNA

## Abstract

**Background:**

According to history, in the pre-Hispanic period, during the conquest and Inka expansion in Ecuador, many Andean families of the Cañar region would have been displaced to several places of *Tawantinsuyu*, including Kañaris, a Quechua-speaking community located at the highlands of the Province of Ferreñafe, Lambayeque (Peru). Other families were probably taken from the Central Andes to a place close to Kañaris, named Inkawasi. Evidence of this migration comes from the presence near the Kañaris–Inkawasi communities of a village, a former Inka camp, which persists until the present day. This scenario could explain these toponyms, but it is still controversial. To clarify this historical question, the study presented here focused on the inference of the genetic relationship between ‘Cañaris’ populations, particularly of Cañar and Ferreñafe, compared to other highland populations. We analysed native patrilineal Y chromosome haplotypes composed of 15 short tandem repeats, a set of SNPs, and maternal mitochondrial DNA haplotypes of control region sequences.

**Results:**

After the genetic comparisons of local populations—three from Ecuador and seven from Peru—, Y chromosome analyses (*n* = 376) indicated that individuals from the Cañar region do not share Y haplotypes with the Kañaris, or even with those of the Inkawasi. However, some Y haplotypes of Ecuadorian ‘Cañaris’ were associated with haplotypes of the Peruvian populations of Cajamarca, Chivay (Arequipa), Cusco and Lake Titicaca, an observation that is congruent with colonial records. Within the Kañaris and Inkawasi communities there are at least five clans in which several individuals share haplotypes, indicating that they have recent common ancestors. Despite their relative isolation, most individuals of both communities are related to those of the Cajamarca and Chachapoyas in Peru, consistent with the spoken Quechua and their geographic proximity. With respect to mitochondrial DNA haplotypes (*n* = 379), with the exception of a shared haplotype of the D1 lineage between the Cañar and Kañaris, there are no genetic affinities.

**Conclusion:**

Although there is no close genetic relationship between the Peruvian Kañaris (including Inkawasi) and Ecuadorian Cañar populations, our results showed some congruence with historical records.

## Background

According to archaeological and anthropological evidence, there were constant migratory events throughout the present Andean territory over the several millennia of the pre-Columbian era, and these probably increased over the last millennium, with the Tiwanaku, Wari and Inka expansions. Inka rulers imposed an important socio-political system of forced displacement and resettlement of people called *mitmakuna*, around the three million square kilometres of the empire (Tawantinsuyu) [1, 2]. During the military campaigns of Tupac Inka Yupanqui and his son Huayna Capac in present day Ecuador, many families from the Cañar region would have been displaced to different parts of Tawantinsuyu [1–4]. Large contingents of military reinforcements were chosen from the subjugated groups to be part of the Inka garrisons, among which were included the Ecuadorian Cañaris [5, 6]. One of the possible settlement sites of *mitmakuna* Cañaris is Kañaris [4], a Quechua-speaking community located in the northern highlands of Peru (Province of Ferreñafe, Lambayeque Department). Next to Kañaris is another Quechua-speaking community called Inkawasi, which would also have been an enclave, but was occupied by *mitmakuna* from southern Peru [7, 8]. Supporting evidence is provided by the chronicles which indicate that near the Inkawasi–Kañaris area was a village, El Ingano, a former Inka camp, which persists until the present, named after Ingano Grande, and located in the district of Sondorillo, Huancabamba, Piura [7, 8].

By 1572, Kañaris was a place of *Reducciones* (Spanish mission reductions) under the political and administration control of Piura. Later, in 1756, some of the Kañaris, Penachí and Salas communities passed into the jurisdiction of the current Inkawasi administration [9]. According to linguistic evidence, the Quechua variant spoken in Kañaris and Inkawasi has been classified as type IIA, the one also spoken in Cajamarca, part of Chachapoyas (Amazonas Department) and highland areas of Piura [10]. Meanwhile, a variant named Quichua or Kichwa is spoken in different regions of Ecuador, including the Province of Cañar, where the ‘Cañaris’ have inhabited since pre-Columbian times [10].

**Fig. 1:**
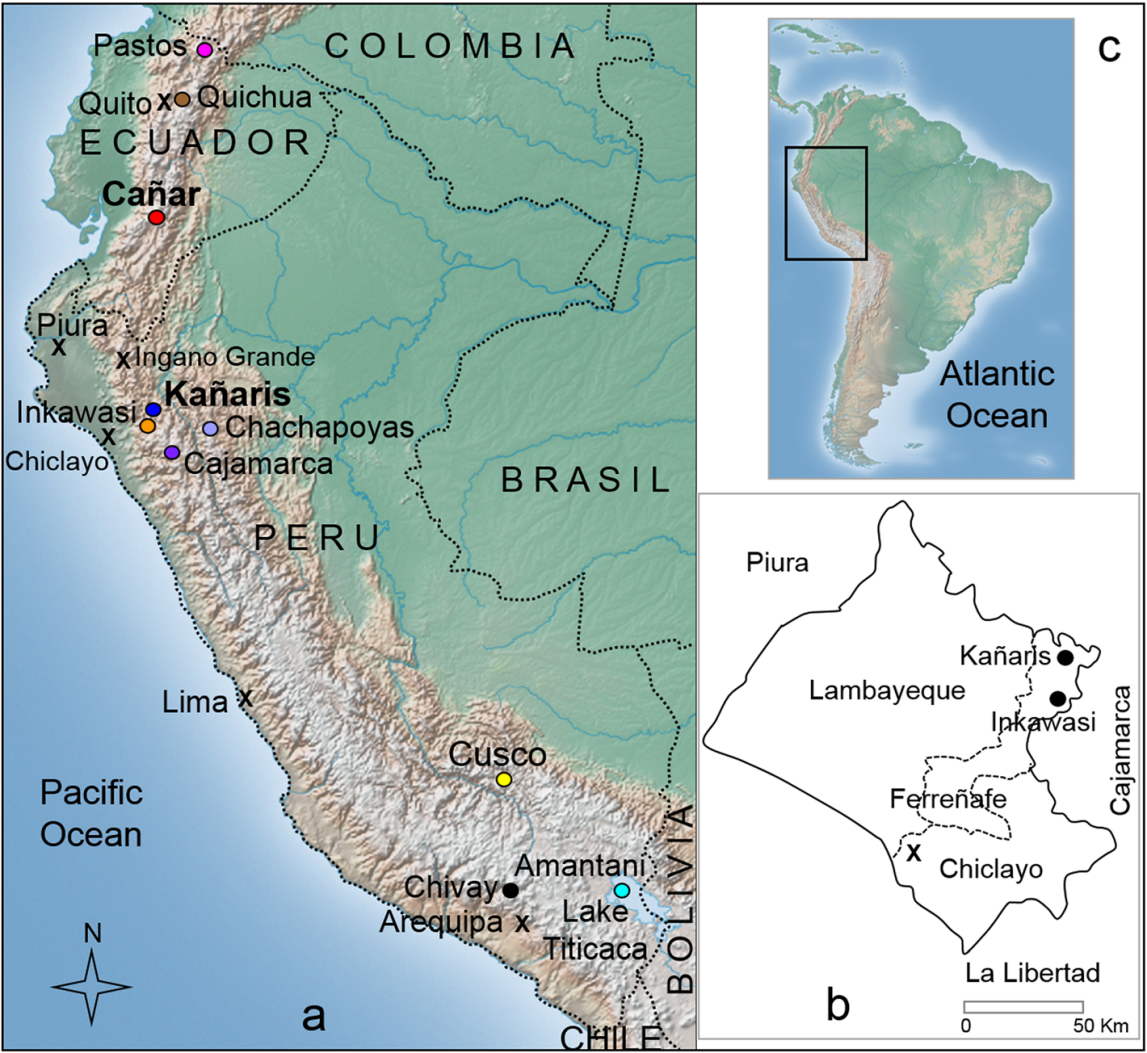
Map of approximately sample locations in the present study. **a** The Ingano Grande community (in Piura Department, Peru) and some reference locations like Province of Ferreñafe (in Lambayeque Department, Peru) are indicated with x symbol. **b** Scheme of Lambayeque Department and its three provinces. **c** Map showing the area of South America where Peru and Ecuador are located (box), including part of other neighboring countries

The general scenario on migration of the people from Cañar region could explain the toponymy in the Province of Ferreñafe (Peru), but this is still controversial [11]. To clarify whether this is the case, the present study investigates whether there is a close genetic relationship between Cañaris and Kañaris, in contrast with other highland populations (Fig. 1). The historical controversies about *mitmakuna* ‘Cañaris’ indicate a very complicated scenario [9, 11], but under the lens of DNA, part of the genealogical history could be elucidated or partially reconstructed [12–16].

## Results and discussion

### Y chromosome

A total of 140 individuals were genotyped and compared with other highland populations of the South American Genographic Project database and published data (*n* = 376) [12–15, 17]. A list of 17 short tandem repeats (STRs) haplotypes of Q-L54 lineages (major native Y chromosome lineage) obtained for the studied populations is presented in Additional file **1**: Table S1a.

**Fig. 2:**
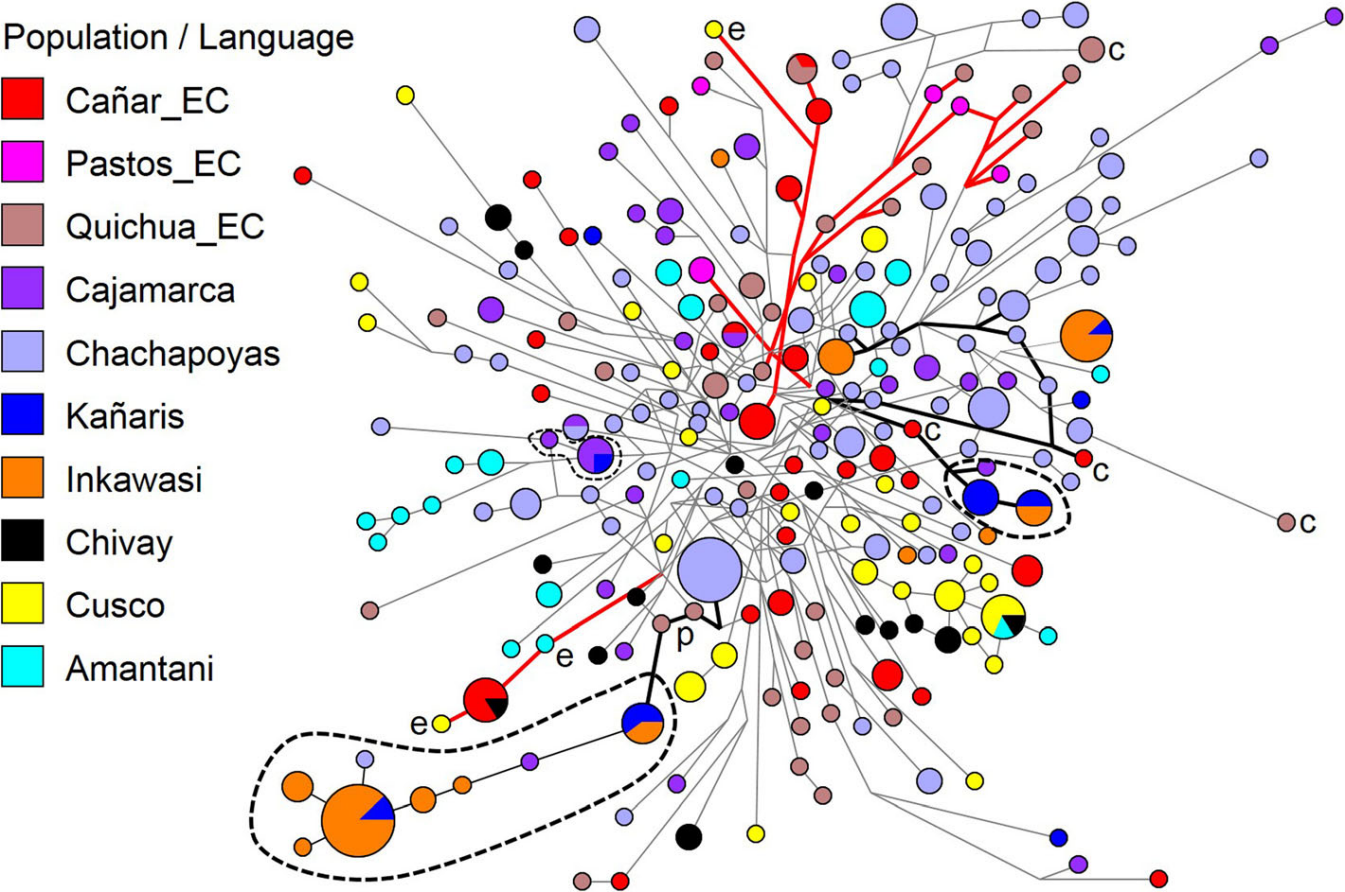
Median joining network for 15 Y-STRs haplotypes among 10 Peruvian and Ecuadorian populations (*n* = 376). The populations are depicted with distinct colours. The haplotypes are represented by circles with sizes proportional to number of individuals, and branch lengths are proportional to STR mutation steps (one step unit between haplotypes in the p). The thick red branches represent connected haplotypes more between many Ecuadorians, while the black branches – Peruvians. The dotted lines point out a cluster of related haplotypes

We used median joining networks to infer the genetic relationships among the STRs haplotypes at the individual level that are shown in Fig. 2. The ‘Cañaris’ of Ecuador are a very heterogeneous group, similar to other Ecuadorian Quichua speakers, showing a considerable diversity of haplotypes (Fig. 2). This scenario fits with the stories suggested in the chronicles: the current Province of Cañar was a conglomerate of several chiefdoms, similar to those of Quito, and its territorial extension included the current region of Piura in Peru [7, 18]. Likewise, the results show that individuals from the Cañar region (Cañar_EC) do not share STR haplotypes with those of the Kañaris and Inkawasi communities. There are some haplotypes of the Ecuadorian ‘Cañaris’ (labelled with the letter **e**) closely related to some from Cajamarca, Chivay (Arequipa), Cusco and Lake Titicaca, an association supported by colonial records. Individuals from the Inkawasi and Kañaris communities are a conglomerate of at least five clans, and most of their haplotypes are shared. Some haplotypes from both localities are more related to those from Cajamarca and Chachapoyas, congruent with the results from linguistic analyses and their geographical proximity [10]. In contrast, there is a close relation between a group of related haplotypes of the Kañaris and Inkawasi and two Quichua-speaking individuals from Ecuador (labelled with the letter p, Fig. 2). However, these two individuals from Ecuador are more related to the Chachapoyas population, and it is probable that their ancestors were *mitmakuna*, brought from the north of Peru to Ecuador, including the individuals labelled with the letter **c** (Fig. 2).

**Fig. 3:**
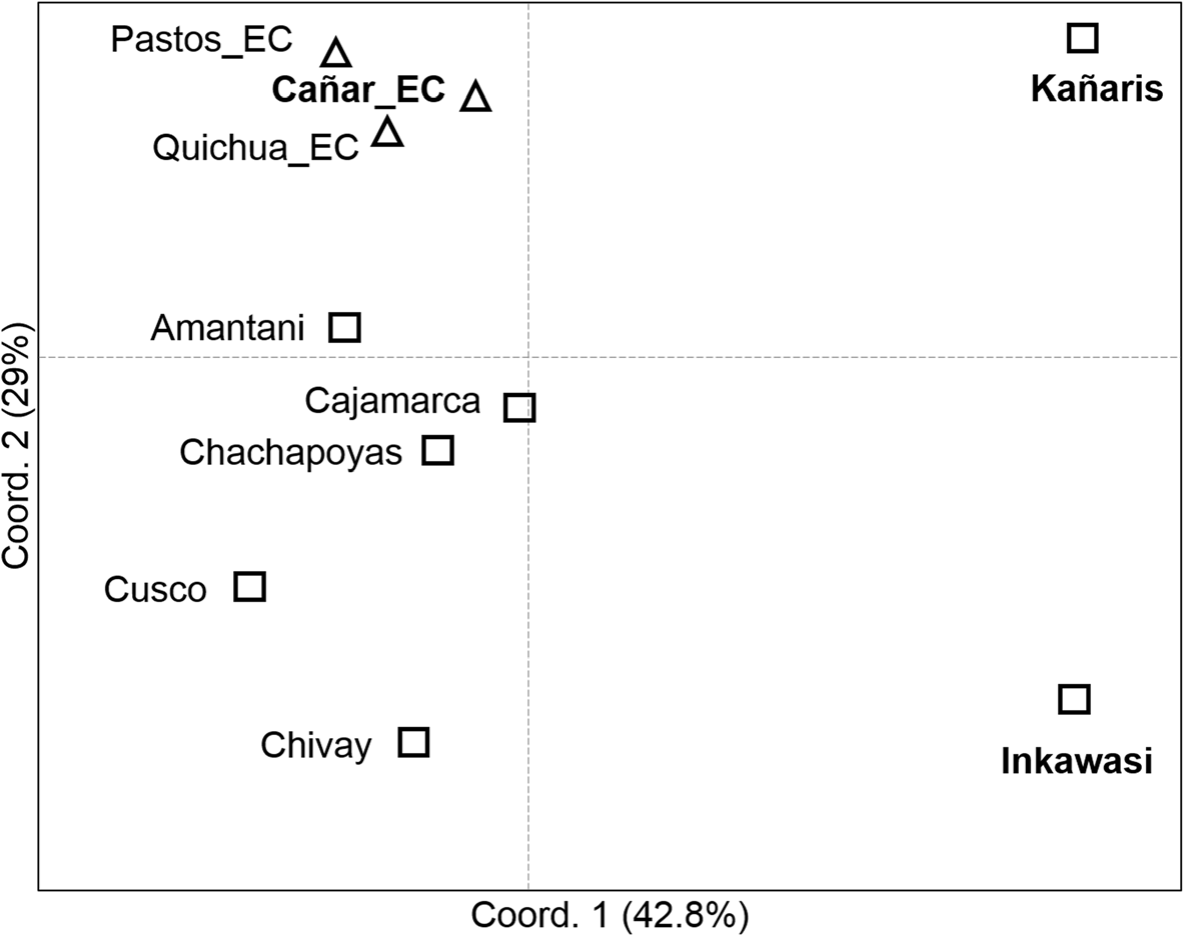
Principal coordinates analysis (PCoA) for Y-STR data among 10 Peruvian and Ecuadorian populations. Reynolds’ *R*_*st*_ coancestry distances were used among the populations. Peruvian populations are represented by squares and Ecuadorian populations by triangles

At the population level, the Analysis of Molecular Variance (AMOVA) results (see Additional file 1: Table S1b), visualised using Principal Coordinates Analysis (PCoA) (Fig. 3), suggest that there is a genetic affinity between the Cañar_EC, Pastos_EC and Quichua_EC. The *R*_*ST*_ index of Cañar with the other two groups is 0.046 (*p* = 0.01504) and 0.047 (*p* = 0.0053), respectively (see Additional file 2: Table S2). This finding is consistent with observations at the individual level. However, due to the low haplotype diversity (h), the populations of the Inkawasi (h = 0.833) and Kañaris (h = 0.908) (see Additional file 3: Table S3) are separated from other populations. This observation makes sense in light of the historical data, which indicate that during the colonial and republic times, both communities, and other nearby ones such as Penachí and Salas, remained in relative geographic isolation [9]. The AMOVA analyses appear to reflect the differences between the 10 populations studied (*R*_*st*_ = 0.099, *p* < 0.001) (see Additional file 1: Table S1b). The *R*_*ST*_ value between Cañar_EC and Kañaris showed moderate difference (*R*_*ST*_ = 0.141, *p* = 0.001). There is also a genetic difference between Kañaris and Inkawasi (*R*_*ST*_ = 0.151, *p* = 0.008). A high level of difference is observed between Cañar_EC and Inkawasi (*R*_*ST*_ = 0.237, *p* < 0.001) (see Additional file 2: Table S2). However, due to the connection of the haplotypes of Kañaris–Inkawasi with other haplotypes from Ecuador (labelled with the letters **p** and **c**, suggested as probable *mitmakuna* from the Peruvian north, particularly from Chachapoyas, Fig. 2), the expected relationship is observed in the PCoA at the population level. Kañaris is more connected to the north highland populations of Peru than to the southern ones. This observation is consistent with the macro regional genomic landscape inferred using a DNA chip with about 630,000 autosomal SNP markers, which showed close genetic relationships between the autochthonous Northern Coast Highland populations from Peru [19]. Kañaris and Cajamarca have been historically associated since the Inka era, in addition to being geographically adjacent [1, 9]. It seems that some of the inhabitants of the Inkawasi community are related to individuals from southern Peru, suggesting that a proportion of their ancestors were brought as *mitmakuna* [7, 8]. Taking into account the geographic isolation and genetic drift, the Inkawasi community was differentiated from the rest of the populations in a similar way to the Kañaris and there are also differences between the two groups (Fig. 3). However, due to cultural issues and similar geographic niches, there was inevitably gene flow between both communities, as shown in the analysis at the individual level (Fig. 2). Another observation arising from the PCoA scatter plot is the genetic affinity between the populations of Cajamarca and Chachapoyas. The *R*_*ST*_ value is 1.3% (*p* = 0.079), a small difference (see Additional file 2: Table S2).

In the Amantani population there is a high haplotype diversity (h = 0.966), despite its geographical isolation on an island located in the Lake Titicaca. Part of the male population of Amantani is associated with the populations of Ecuador, Cajamarca, Chachapoyas and Cusco, suggesting that considerable gene flow took place among the Andean communities. However, the macro regional scenario depicted in the PCoA space, with the exception of Amantani, reflects a genetic gradient according to geographic location.

### Mitochondrial DNA

A total of 182 mitochondrial DNA (mtDNA) control region haplotypes were assigned to four Native American maternal lineages (A2, B2, C1 and D1) and compared with other highland populations as done previously with the STR haplotypes. Genetic analysis was carried out using only the autochthonous lineages (*n* = 379). A list of the mtDNA control region data according to the rCRS is presented in Additional file 4: Table S4a. With some exceptions, the distribution of haplogroup frequencies showed that B2 is the most frequent lineage among the highland populations (see Additional file 4: Table S4c).

**Fig. 4:**
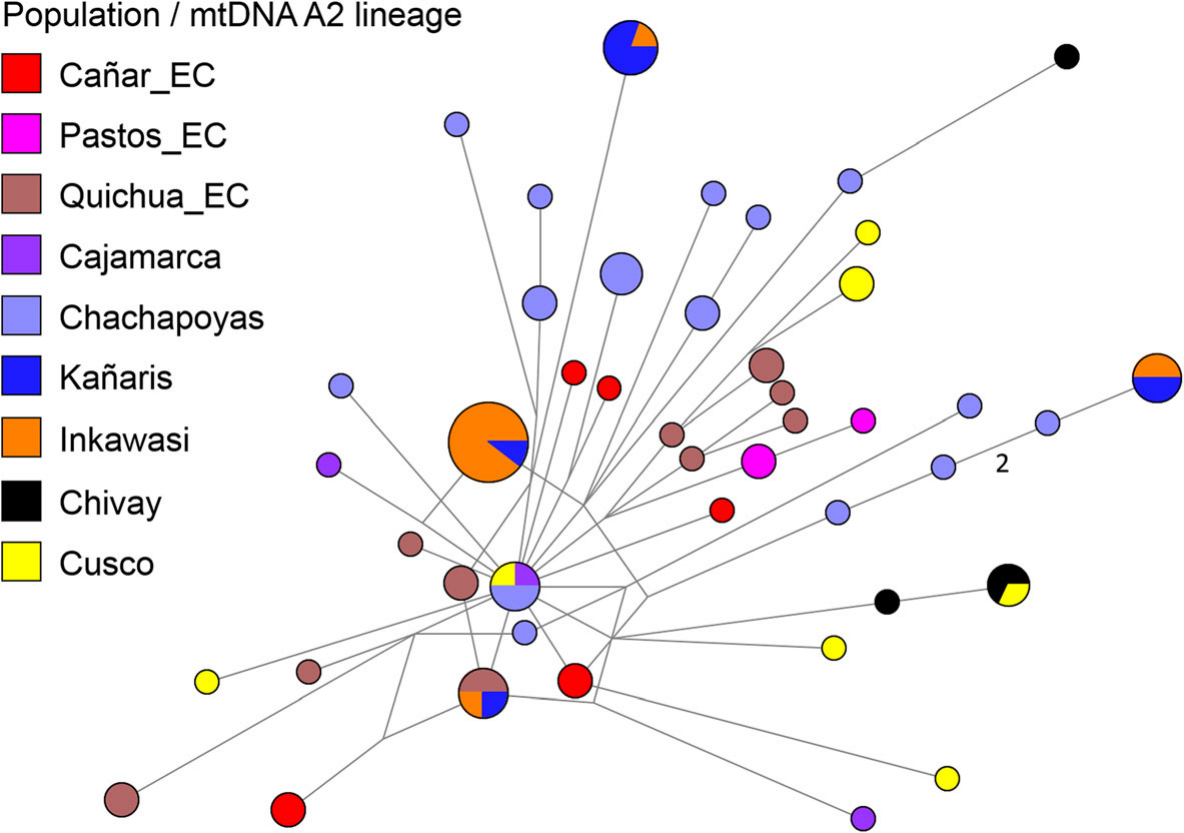
Median joining network for mtDNA haplotypes of A2 lineage among Peruvian and Ecuadorian populations (*n* = 80). The populations are depicted with distinct colours. The mitochondrial haplotypes are represented by circles with sizes proportional to numbers of individuals, and branch lengths are proportional to mutation steps (for example, two nucleotide changes indicated in the figure)

**Fig. 5:**
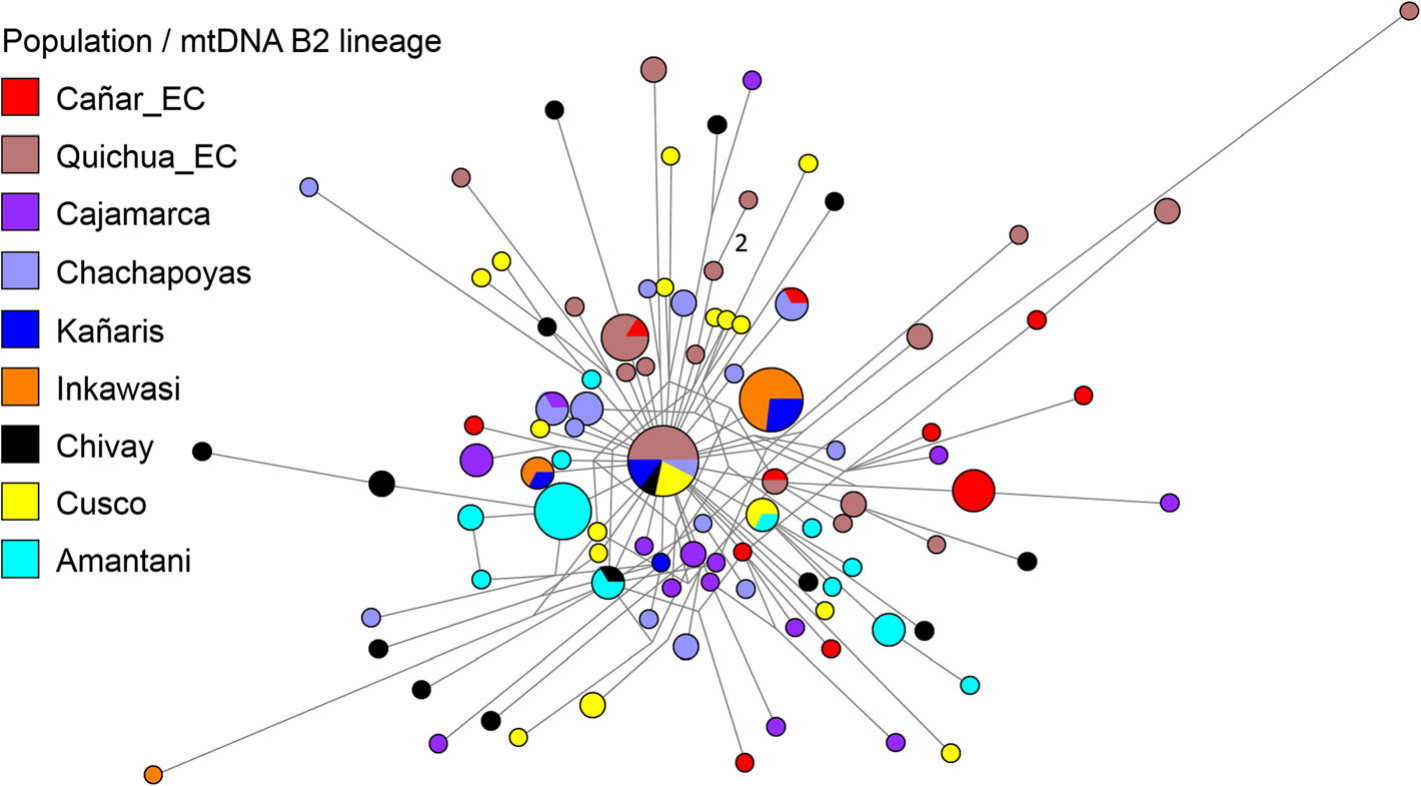
Median joining network for mtDNA haplotypes of B2 lineage among Peruvian and Ecuadorian populations (*n* = 163). The populations are depicted with distinct colours. The mitochondrial haplotypes are represented by circles with sizes proportional to numbers of individuals, and branch lengths are proportional to mutation steps (for example, two nucleotide changes indicated in the figure)

**Fig. 6:**
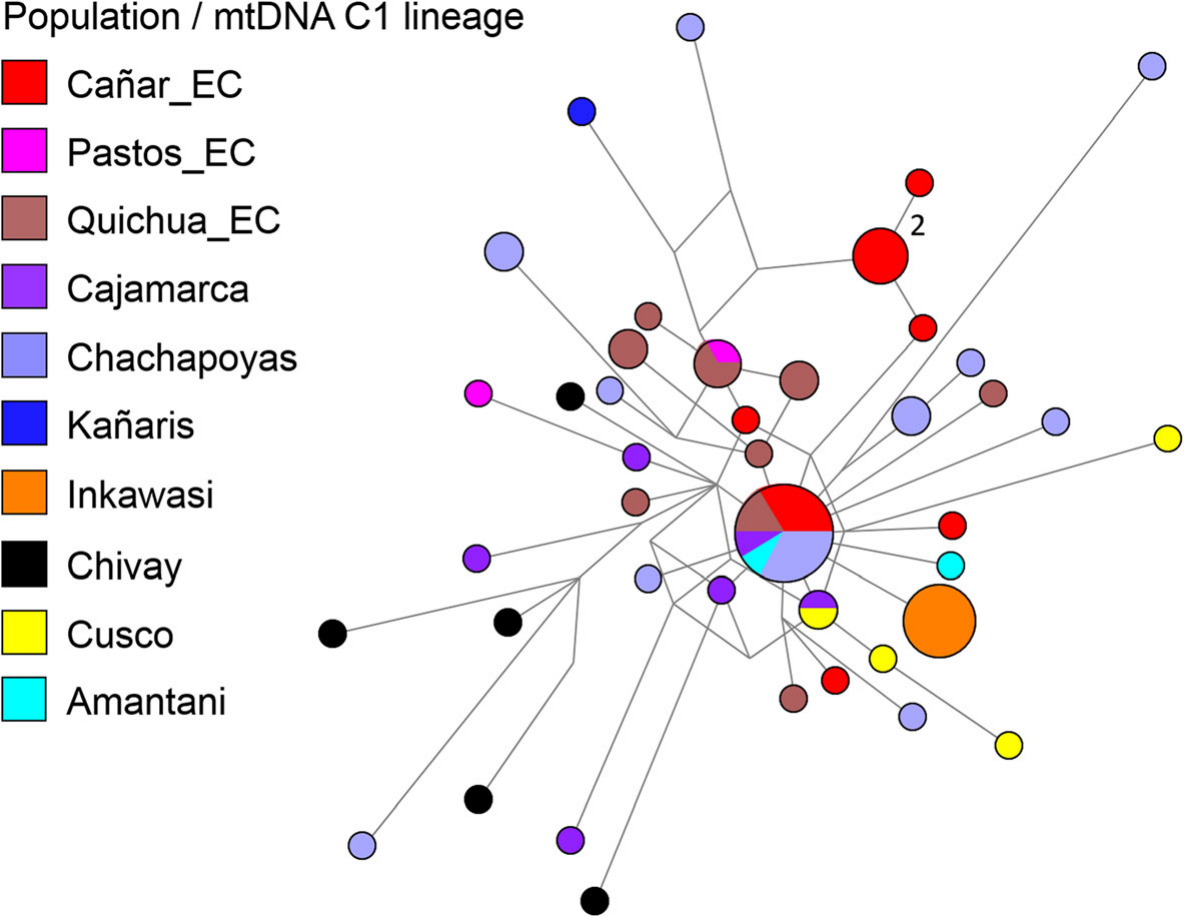
Median joining network for mtDNA haplotypes of C1 lineage among Peruvian and Ecuadorian populations (*n* = 69). The populations are depicted with distinct colours. The mitochondrial haplotypes are represented by circles with sizes proportional to numbers of individuals, and branch lengths are proportional to mutation steps (for example, two nucleotide changes indicated in the figure)

**Fig. 7:**
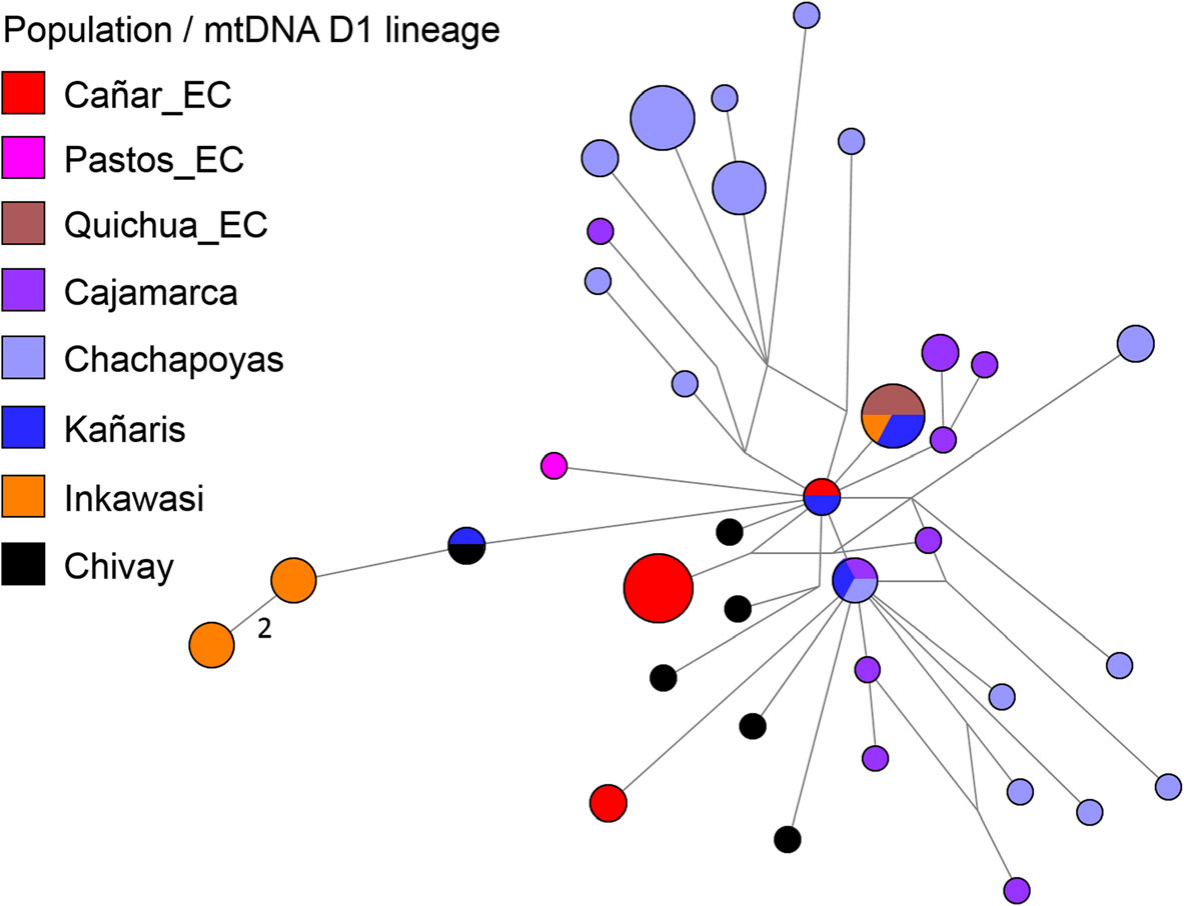
Median joining network for mtDNA haplotypes of D1 lineage among Peruvian and Ecuadorian populations (*n* = 67). The populations are depicted with distinct colours. The mitochondrial haplotypes are represented by circles with sizes proportional to numbers of individuals, and branch lengths are proportional to mutation steps (for example, two nucleotide changes indicated in the figure)

**Fig. 8:**
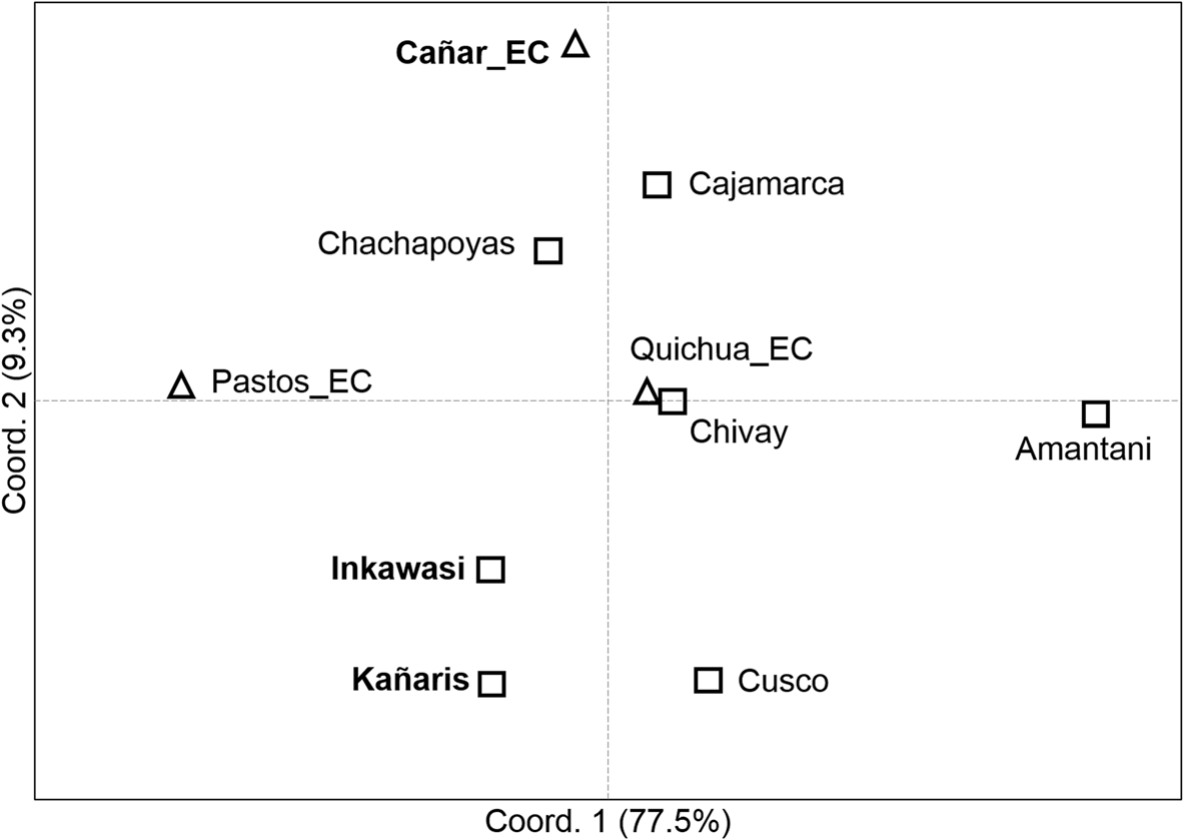
Principal coordinates analysis (PCoA) for mtDNA data among 10 Peruvian and Ecuadorian populations. Reynolds’ *ϕ*_*st*_ genetic distances were used among populations. Peruvian populations are represented by squares and Ecuadorian populations by triangles

At an individual level, we inferred phylogenetic relationships using median joining networks, as with the paternal line. According to the results shown in Figs. 4, 5, 6 and 7, with the exception of one haplotype shared by the D1 lineage between Cañar and Kañaris, there are no genetic affinities between these populations. However, this is not the case when comparing Kañaris and Quichua_EC, where some haplotypes within the A2, B2 and D1 lineages are shared. Most of the Inkawasi and Kañaris individuals share haplotypes, corresponding to lineages A2, B2 and D1. In the case of lineage C1, there is a shared haplotype between several individuals from Cañar, Quichuas from Ecuador, northern Peru (Chachapoyas and Cajamarca) and Amantani (Lake Titicaca), with the exception of Inkawasi and Kañaris. The low *F*_*ST*_ value calculated using AMOVA for the 10 studied populations (*F*_*ST*_ = 0.058, *p* < 0.001 (see Additional file 4: Table S4b), indicates that there is a little difference between them. The *ϕ*_*st*_ genetic distances (see Additional file 5: Table S5), visualised in a two-dimensional space such as PCoA (Fig. 8), indicate that the Cañar_EC population is different from the communities of Kañaris and Inkawasi, located at the opposite side, reflecting the correlation between the genetic background and the geographic isolation. Statistical analysis showed a decrease in maternal haplotype diversity (see Additional file 6: Table S6) in the Inkawasi (h = 86.8%), Amantani (h = 87.4%), Pastos_EC (h = 93.3%), Kañaris (h = 94.3%) and Cañar_EC populations (h = 95.3%). Likewise, there is a maternal genetic affinity between the Kañaris and Inkawasi communities (*ϕ*_*st*_ = 0.0067, *p* = 0.292) (Fig. 5); our inference coincides with colonial records, which reported that both groups have been intimately associated since pre-Columbian times until the present [9]. Certainly, the access through the *Qhapaq Ñan*, or great Inka road, that crosses the Kañaris and Inkawasi localities, would have contributed, on the one hand, to the maintenance of the genetic affinity between them, and on the other hand, to their differences, reflecting the admixture of two groups of maternal founders in both the Inkawasi and Kañaris of Lambayeque. One group would be associated with the north of Peru and the other with south Peru. However, the observation of shared mtDNA haplotypes among the populations studied indicates that considerable gene flow took place in the Tawantinsuyu over generations [12, 13].

With respect to the historical association of the Cañar region (Ecuador) with Kañaris of Lambayeque (Peru), it is likely that at the beginning of the colonial period and after the collapse of the Inka Empire, most *mitmakuna* (or their descendants) from Tawantinsuyu, especially from Cañar, returned to their original regions. It is known that a proportion of the Ecuadorian Cañaris, including other groups subjugated by the Inkas, were allied with the Spaniards. This situation would have offered a chance to free them from the Inkas, and also to reintegrate the *mitmakuna* Cañaris and return them to their old home [5]. This could be the reason why we do not detect the presence of Ecuadorian male DNA in the Kañaris community, assuming that the Inkas displaced them to Lambayeque Department. A different situation would have occurred with other Ecuadorian Cañaris, part of the Inka contingent, who would have stayed in their original locations, such as in Cajamarca, Chivay (Arequipa) Cusco and Lake Titicaca. The general scenario shows that the genetic pattern of the individuals associated with the aforementioned places reflect the migrations that took place during the Inka Empire throughout the Andes. Data from ancient DNA of the ‘Cañaris’ from Ecuador as well as from Peru will undoubtedly be the key to complement their history.

## Conclusion

To clarify a historical controversy on displacements of the people during the Inka Empire, we inferred the genetic history of the ‘Cañaris’ populations. Although there is no close genetic relationship between the Peruvian Kañaris (including Inkawasi) and Ecuadorian Cañar populations, our results showed some congruence with historical records.

## Methods

### Participants and sampling

Ethical consent for the project (Genographic Project –South America) was obtained from the Ethics Committees of the following institutions: Universidade Federal de Minas Gerais and CONEP (Brazil), Universidad de las Americas (Ecuador) and Universidad de San Martín de Porres (Peru). The project was previously explained to the confederations, authorities or leaders, as well as to the participants. After informed consent was obtained, saliva samples from each volunteer from different families was collected using buccal swabs. Subsequently, in the laboratory, DNA extraction was performed following standard procedures [12].

For the STR haplotype analysis (*n* = 376), we tested the following 10 populations (Fig. 1): Cañar_EC (*n* = 46), from the Sisid, Zhud and Cuchucun communities, Province of Cañar, Ecuador; Pastos_EC (*n* = 6), from the Province of Carchi, Ecuador; Kañaris (*n* = 16) and Inkawasi (*n* = 39) from the Province of Ferreñafe, Lambayeque, Peru and Chivay (*n* = 17) from of Province of Caylloma, Arequipa, Peru. For comparison, individuals from Chachapoyas (*n* = 115) [15, 17], Cajamarca (*n* = 36) [12, 17], including *n* = 11 in the present study, Quichua_EC (*n* = 37) [13], including *n* = 4 new samples, Cusco (*n* = 38) [12], including *n* = 2 in the present study, and Amantani (*n* = 26) [12] were included.

For mtDNA analysis (*n* = 379), we considered the following populations/languages: Cañar_EC (*n* = 45, from the mentioned communities, Province of Cañar, Ecuador), Pastos_EC (*n* = 6, from the Province of Carchi, Ecuador), Quichua_EC (*n* = 62) [13], including 25 new samples; Cajamarca (*n* = 36) [12], including 17 new samples, Inkawasi (*n* = 38), Kañaris (*n* = 21), Chivay (*n* = 30), Cusco (*n* = 33), Amantani (*n* = 26) [12] and Chachapoyas (*n* = 82) [15].

### Statistical analysis

For the Y chromosome, five SNPs were genotyped, corresponding to the C-M130, Q-M242, Q-M346, Q-L54 and Q-M3 markers, which characterise the autochthonous lineages of America [20], using a TaqMan system Real Time Polymerase Chain Reaction (PCR) in the 7900HT (ABI). Next, only the individuals belonging to the Q-L54 lineages (Q-M3, Q-L54*) were selected for the genotyping with a set of 17 STRs (Y-filer; ABI). For the PCR reactions, a standard protocol was used according to [12] and the amplified products were injected into a capillary electrophoresis system (ABI3130XL Genetic Analyzer equipment; ABI). Next, using the GeneMapper ID v3.2 program (Applied Biosystems, Foster City, California, USA), the STR alleles were determined. The DYS389b variants were determined by subtracting the DYS389II-DYS389I alleles and excluding the DYS385 marker in the statistical analyses. For control region mtDNA, the PCR reactions and amplicon sequencing were carried out following a previously published procedure [12] using the ABI3130XL Genetic Analyzer (ABI). For the analysis of mtDNA sequences, the SeqScape v2.6 program (Applied Biosystems) and alignments according to rCRS reference [21] were used. Mitochondrial haplogroups were determined using the prediction program MitoTool [22]. Due to discrepancies in the alignments, the mitochondrial sequences at the positions 303–315, 515–522, 16,182–16,193 and 16,519 were not considered in the statistical analyses. To elucidate the genetic relationships between haplotypes, the Network 5.0.1.0 program was used (**http://www.fluxus-engineering.com**) with the single-stepwise mutation model and median joining algorithm as well as the maximum parsimony approach [23, 24]. A weighting criterion was followed for the STRs, as described by [12]. To infer genetic relationships at the population level, the genetic diversity indices F_ST_ (*R*_*ST*_ for STRs and *ϕ*_*st*_ for mtDNA) and genetic distances between the 10 populations of Peru and Ecuador were evaluated using the AMOVA algorithm, implemented in Arlequin 3.5.1.2. program [25]. The haplotype diversity indices and the neutrality tests for the mtDNA were estimated taking into account 10,000 permutations. For the PCoA, the program GenAlEx v6.5 (**http://biology.anu.au/GenAlEx**) was used, considering the genetic distance indices transformed by the Reynolds formula in the Arlequin program.

## Supplementary information

**Additional file 1: Table S1.** Y-STR data and AMOVA results. (**a**) List of 17 Y-STRs among Peruvian and Ecuadorian populations; (**b**) AMOVA results for the 10 Peruvian and Ecuadorian populations using 15 STR haplotypes of Y chromosome (*n* = 376).

**Additional file 2: Table S2.** The *R*_*ST*_ matrix (lower diagonal) and their statistical probabilities (upper diagonal) among the 10 populations, according to Arlequin v3.5.1.2 program, based on the 15 Y-STR haplotypes.

**Additional file 3: Table S3.** Diversity indices for 15 the Y-STR haplotypes among 10 populations studied. n = sample size; K = number of haplotypes; N Y-15 STRs = number of 15 Y-STR polymorphic markers; *h* = haplotypic diversity; *MPD* = mean number of pairwise differences; SD = standard deviation.

**Additional file 4: Table S4.** MtDNA SNPs data, AMOVA results and mtDNA haplogroup frequencies. **a** List of mtDNA SNPs according to rCRS among the Peruvian and Ecuadorian populations. The mitochondrial haplogroups were determined using MitoTool program (http://mitotool.kiz.ac.cn/); **b** AMOVA results for the 10 Peruvian and Ecuadorian populations using the control region mtDNA sequences (*n* = 379); **c** Distribution of mtDNA haplogroup frequencies among Ecuadorian and Peruvian populations.

**Additional file 5: Table S5.** The *ϕ*_*st*_ matrix (lower diagonal) and their statistical probabilities (upper diagonal) among 10 populations, according to Arlequin v3.5.1.2. program, based on control region mtDNA analysis.

**Additional file 6: Table S6.** Diversity indices and neutrality tests for each population using 379 mitochondrial sequences of the control region for the four maternal lineages (A2, B2, C1, D1). n = sample size; K = number of haplotypes; *h* = haplotypic diversity; S = number of polymorphic sites; π = nucleotide diversity (average over all loci); *D* = Neutrality Tajima’s D test; *Fs* = Fu’s Fu test; *p* = probability.

## Data Availability

The STRs and mtDNA data supporting the conclusions are available in the Additional file 1: Table S1a and Additional file 4: Table S4a. MtDNA control region sequences of 182 individuals have been deposited in NCBI GenBank under accession numbers MN707742-MN707923.

## Abbreviations

STRs: Short tandem repeats
SNP: Single nucleotide polymorphism
mtDNA: Mitochondrial DNA
AMOVA: Analysis of Molecular Variance
PCoA: Principal Coordinates Analysis
rCRS: Revised Cambridge Reference Sequence
