# Supplementary material for: Tracing the genetic history of the ‘Cañaris’ from Ecuador and Peru using uniparental DNA markers
Source: BMC Genomics. 2020 Sep 10;21(Suppl 7):413. doi: 10.1186/s12864-020-06834-1 (PMC7488242; doi:10.1186/s12864-020-06834-1)
Supplement: Supplementary file 2 — Additional file 2: Table S2. The RST matrix (lower diagonal) and their statistical probabilities (upper diagonal) among the 10 populations, according to Arlequin v3.5.1.2 program, based on the 15 Y-STR haplotypes. [file 12864_2020_6834_MOESM2_ESM.docx]

| Cañar_EC  1 | Pastos_EC  2 | Quichua_EC  3 | Cajamarca  4 | Chachapoyas  5 | Kañaris  6 | Inkawasi  7 | Chivay  8 | Cusco  9 | Amantani  10 |  |
| --- | --- | --- | --- | --- | --- | --- | --- | --- | --- | --- |
| 0 | 0.15038+-0.0037 | 0.00535+-0.0008 | 0.00812+-0.0009 | 0.00000+-0.0000 | 0.00109+-0.0003 | 0.00000+-0.0000 | 0.00178+-0.0004 | 0.00020+-0.0001 | 0.00158+-0.0004 | 1 |
| 0.04608 | 0 | 0.52965+-0.0054 | 0.06098+-0.0023 | 0.02723+-0.0014 | 0.01950+-0.0014 | 0.01505+-0.0012 | 0.01040+-0.0010 | 0.08306+-0.0027 | 0.35452+-0.0046 | 2 |
| 0.04732 | 0 | 0 | 0.01614+-0.0013 | 0.00000+-0.0000 | 0.00020+-0.0001 | 0.00000+-0.0000 | 0.00010+-0.0001 | 0.00188+-0.0004 | 0.16295+-0.0041 | 3 |
| 0.04007 | 0.0821 | 0.03481 | 0 | 0.07940+-0.0026 | 0.00109+-0.0003 | 0.00000+-0.0000 | 0.03772+-0.0019 | 0.00287+-0.0005 | 0.08504+-0.0029 | 4 |
| 0.06495 | 0.1044 | 0.07418 | 0.01354 | 0 | 0.00000+-0.0000 | 0.00000+-0.0000 | 0.00218+-0.0005 | 0.00000+-0.0000 | 0.00010+-0.0001 | 5 |
| 0.14082 | 0.19848 | 0.1558 | 0.13969 | 0.2236 | 0 | 0.00772+-0.0008 | 0.00040+-0.0002 | 0.00000+-0.0000 | 0.00050+-0.0002 | 6 |
| 0.23754 | 0.24563 | 0.22171 | 0.14192 | 0.19018 | 0.15099 | 0 | 0.00287+-0.0006 | 0.00000+-0.0000 | 0.00000+-0.0000 | 7 |
| 0.11108 | 0.1494 | 0.10078 | 0.04108 | 0.07535 | 0.24803 | 0.15694 | 0 | 0.34224+-0.0045 | 0.03990+-0.0020 | 8 |
| 0.10681 | 0.0809 | 0.06156 | 0.05972 | 0.09248 | 0.27456 | 0.2213 | 0.00325 | 0 | 0.12504+-0.0029 | 9 |
| 0.08992 | 0.0063 | 0.01412 | 0.02485 | 0.08298 | 0.18958 | 0.20039 | 0.05664 | 0.02119 | 0 | 10 |

**Table S2**. *R_ST_* matrix (lower diagonal) and their statistical probabilities (upper diagonal) among 10 populations, according to Arlequin v3.5.1.2 program, based on the 15 Y-STR haplotypes.
