# Supplementary material for: Tracing the genetic history of the ‘Cañaris’ from Ecuador and Peru using uniparental DNA markers
Source: BMC Genomics. 2020 Sep 10;21(Suppl 7):413. doi: 10.1186/s12864-020-06834-1 (PMC7488242; doi:10.1186/s12864-020-06834-1)
Supplement: Supplementary file 3 — Additional file 3: Table S3. Diversity indices for 15 the Y-STR haplotypes among 10 populations studied. n = sample size; K = number of haplotypes; N Y-15 STRs = number of 15 Y-STR polymorphic markers; h = haplotypic diversity; MPD = mean number of pairwise differences; SD = standard deviation. [file 12864_2020_6834_MOESM3_ESM.docx]

| Population | n | K | N 15  Y-STRs | *h*  (SD) | *MPD*  (SD) |
| --- | --- | --- | --- | --- | --- |
| Cañar_EC | 46 | 30 | 15 | 0.9739 (0.011) | 7.145894 (3.413) |
| Pastos_EC | 6 | 5 | 13 | 0.9333 (0.122) | 7.933333 (4.302) |
| Quichua_EC | 37 | 33 | 15 | 0.994 (0.008) | 7.481982 (3.576) |
| Cajamarca | 36 | 30 | 15 | 0.9731 (0.016) | 6.615385 (3.190) |
| Chachapoyas | 115 | 76 | 15 | 0.982 (0.006) | 6.982609 (3.305) |
| Kañaris | 16 | 9 | 14 | **0.9083** (0.048) | 6.658333 (3.317) |
| Inkawasi | 39 | 12 | 13 | **0.8327** (0.045) | 4.916329 (2.447) |
| Chivay | 17 | 14 | 14 | 0.9779 (0.027) | 6.786765 (3.364) |
| Cusco | 38 | 28 | 14 | 0.9787 (0.012) | 6.411095 (3.104) |
| Amantani | 26 | 18 | 13 | 0.9662 (0.021) | 6.070769 (2.986) |

**Table S3**. Diversity indices for 15 the Y-STR haplotypes among 10 populations studied. n = sample size; K = number of haplotypes; N 15 Y-STRs = number of 15 Y-STR polymorphic markers; *h* = haplotypic diversity; *MPD* = mean number of pairwise differences; SD = standard deviation.
