# Supplementary material for: Tracing the genetic history of the ‘Cañaris’ from Ecuador and Peru using uniparental DNA markers
Source: BMC Genomics. 2020 Sep 10;21(Suppl 7):413. doi: 10.1186/s12864-020-06834-1 (PMC7488242; doi:10.1186/s12864-020-06834-1)
Supplement: Supplementary file 5 — Additional file 5: Table S5. The ϕst matrix (lower diagonal) and their statistical probabilities (upper diagonal) among 10 populations, according to Arlequin v3.5.1.2. program, based on control region mtDNA analysis. [file 12864_2020_6834_MOESM5_ESM.docx]

| Cañar  1 | Pastos  2 | Quichua  3 | Cajamarca  4 | Chachapoyas  5 | Kañaris  6 | Inkawasi  7 | Chivay  8 | Cusco  9 | Amantani  10 |  |
| --- | --- | --- | --- | --- | --- | --- | --- | --- | --- | --- |
| 0 | 0.02851+-  0.0016 | 0.03911+-  0.0019 | 0.05722+-  0.0024 | 0.01634+-  0.0012 | 0.01079+-  0.0011 | 0.01208+-  0.0012 | 0.06108+-  0.0023 | 0.00089+-  0.0003 | 0.00000 | 1 |
| 0.12128 | 0 | 0.03039+-  0.0018 | 0.00465+-  0.0007 | 0.05574+-  0.0021 | 0.07712+-  0.0025 | 0.12375+-  0.0030 | 0.01584+-0.0012 | 0.00822+-0.0009 | 0.00000 | 2 |
| 0.02407 | 0.13468 | 0 | 0.05267+-  0.0023 | 0.00178+-  0.0005 | 0.02911+-  0.0015 | 0.00782+-  0.0010 | 0.2700+-0.0038 | 0.11415+-0.0032 | 0.00030+-0.0002 | 3 |
| 0.02315 | 0.18434 | 0.02368 | 0 | 0.01257+-  0.0012 | 0.01624+-  0.0012 | 0.00376+-  0.0006 | 0.34531+-0.0048 | 0.02138+-0.0013 | 0.00010+-0.0001 | 4 |
| 0.0258 | 0.07539 | 0.03634 | 0.0295 | 0 | 0.05445+-  0.0025 | 0.01594+-  0.0012 | 0.03099+-0.0017 | 0.00030+-0.0002 | 0.00000 | 5 |
| 0.06414 | 0.09866 | 0.04733 | 0.05936 | 0.02802 | 0 | 0.29185+-  0.0046 | 0.08197+-0.0028 | 0.02376+-0.0017 | 0.00000 | 6 |
| 0.04562 | 0.0659 | 0.04787 | 0.06479 | 0.03223 | **0.0067** | 0 | 0.03089+-0.0016 | 0.00406+-0.0006 | 0.00000 | 7 |
| 0.02413 | 0.1434 | 0.00524 | 0.0015 | 0.02466 | 0.02967 | 0.03928 | 0 | 0.13316+-0.0033 | 0.00079+-0.0003 | 8 |
| 0.07621 | 0.19782 | 0.01605 | 0.04275 | 0.07445 | 0.05946 | 0.07683 | 0.01654 | 0 | 0.00050+-0.0002 | 9 |
| 0.22189 | 0.69259 | 0.12461 | 0.16152 | 0.20647 | 0.30338 | 0.2715 | 0.11316 | 0.10903 | 0 | 10 |

**Table S5**. The *φ_st_* matrix (lower diagonal) and their statistical probabilities (upper diagonal) among 10 populations, according to Arlequin v3.5.1.2. program, based on control region mtDNA analysis.
