# Supplementary material for: Tracing the genetic history of the ‘Cañaris’ from Ecuador and Peru using uniparental DNA markers
Source: BMC Genomics. 2020 Sep 10;21(Suppl 7):413. doi: 10.1186/s12864-020-06834-1 (PMC7488242; doi:10.1186/s12864-020-06834-1)
Supplement: Supplementary file 6 — Additional file 6: Table S6. Diversity indices and neutrality tests for each population using 379 mitochondrial sequences of the control region for the four maternal lineages (A2, B2, C1, D1). n = sample size; K = number of haplotypes; h = haplotypic diversity; S = number of polymorphic sites; π = nucleotide diversity (average over all loci); D = Neutrality Tajima’s D test; Fs = Fu’s Fu test; p = probability. [file 12864_2020_6834_MOESM6_ESM.docx]

| Population | n | Diversity indices | | | | Neutrality tests | | | |
| --- | --- | --- | --- | --- | --- | --- | --- | --- | --- |
|  |  | K | *h* | S | π | *D* | *p* | *Fs* | *p* |
| Cañar_EC | 45 | 26 | **0.9535** | 53 | 0.009611 | -0.67386 | 0.28 | -5.10066 | 0.068 |
| Pastos_EC | 6 | 5 | **0.9333** | 28 | 0.012759 | 0.73158 | 0.78 | 1.3684 | 0.68 |
| Quichua_EC | 62 | 38 | 0.9757 | 75 | 0.010024 | -1.08129 | 0.132 | -13.2046 | 0.002 |
| Cajamarca | 36 | 32 | 0.9921 | 66 | 0.00931 | -1.41663 | 0.058 | -19.9172 | 0 |
| Chachapoyas | 82 | 56 | 0.9868 | 100 | 0.011063 | -1.40096 | 0.053 | -24.3391 | 0 |
| Kañaris | 21 | 13 | **0.9429** | 38 | 0.009506 | 0.14763 | 0.608 | -0.51045 | 0.428 |
| Inkawasi | 38 | 11 | **0.8677** | 44 | 0.01117 | 0.55250 | 0.763 | 5.40756 | 0.955 |
| Chivay | 30 | 28 | 0.9954 | 80 | 0.01088 | -1.64238 | 0.030 | -15.6125 | 0 |
| Cusco | 33 | 28 | 0.9886 | 65 | 0.009633 | -1.31384 | 0.073 | -13.6001 | 0.001 |
| Amantani | 26 | 14 | **0.8738** | 28 | 0.004001 | -1.46633 | 0.054 | -3.92703 | 0.047 |

**Table S6**. Diversity indices and neutrality tests for each population using 379 mitochondrial sequences of the control region for the four maternal lineages (A2, B2, C1, D1). n = sample size; K = number of haplotypes; *h* = haplotypic diversity; S = number of polymorphic sites; π = nucleotide diversity (average over all loci); *D* = Neutrality Tajima’s D test; *Fs* = Fu’s Fu test; *p* = probability.
